# Investigation of New Microstrip Bandpass Filter Based on Patch Resonator with Geometrical Fractal Slot

**DOI:** 10.1371/journal.pone.0152615

**Published:** 2016-04-07

**Authors:** Yaqeen S. Mezaal, Halil T. Eyyuboglu

**Affiliations:** Electronic and Communication Engineering Department, Cankaya University, Ankara, Turkey; Drexel University, UNITED STATES

## Abstract

A compact dual-mode microstrip bandpass filter using geometrical slot is presented in this paper. The adopted geometrical slot is based on first iteration of Cantor square fractal curve. This filter has the benefits of possessing narrower and sharper frequency responses as compared to microstrip filters that use single mode resonators and traditional dual-mode square patch resonators. The filter has been modeled and demonstrated by Microwave Office EM simulator designed at a resonant frequency of 2 GHz using a substrate of ε_r_ = 10.8 and thickness of h = 1.27 mm. The output simulated results of the proposed filter exhibit 22 dB return loss, 0.1678 dB insertion loss and 12 MHz bandwidth in the passband region. In addition to the narrow band gained, miniaturization properties as well as weakened spurious frequency responses and blocked second harmonic frequency in out of band regions have been acquired. Filter parameters including insertion loss, return loss, bandwidth, coupling coefficient and external quality factor have been compared with different values of perturbation dimension (d). Also, a full comparative study of this filter as compared with traditional square patch filter has been considered.

## Introduction

Traditional microstrip bandpass filters are mostly constructed using single-mode resonators. Recently, dual-mode resonators have been widely adopted in the microwave and RF wireless communication applications for their high performances and low loss properties. Since they have double resonant nature, a dual-mode bandpass filter of some order requires half as multiple resonators as compared with traditional topology [1]. Dual-mode standard for planar resonators is distinguished and has been the area under discussion of wide studies before more than four decades [2]. This dual mode theory is distinctive to all symmetrical resonators that are able to degenerate modes with the same resonant frequency. When the symmetry is kept intact, the two modes are orthogonal and they cannot exchange the microwave power. On the contrary, when the geometrical symmetry is opportunely broken, the resonator boundary conditions change allowing the coupling between the modes. Consequently, two modes can be contemporarily present at slightly split frequencies. In fact, the most important benefit of dual-mode resonator is that each resonator can be used as dual tuned resonant circuit, and hence the required number of resonators for n-order filter is decreased by a half, in form of a miniaturized filter configuration [1–2].

Microstrip dual-mode filter is originally initiated by Wolff [2] in 1972. Many compact dual-mode bandpass filters such as square loop [3], meander loop [4], circular ring [5], patch based resonators [6] and open loop resonators [7] have already been reported in the literature as doubly tuned degenerate mode filters.

Non-degenerate dual-mode filters have been introduced as stated in[8–10] with higher bandwidth up to 25% as compared to degenerate dual mode filters with usual narrow bandwidth of (< %5).

However, there are still much research interests concerning the development of more compact dual mode filters with various electrical specifications [11–12].

In this paper, dual degenerate mode microstrip slotted patch bandpass filter with narrow band responses and sufficient performances has been presented. The proposed filter uses geometrical slot based on Cantor square fractal curve applied to square patch resonator. A parametric study about the effect of perturbation side length (d) on the slotted bandpass filter in terms of electrical parameters has been extracted. Also, a comparative investigation into microstrip bandpass filters using and without using geometrical slot has been achieved in this research article using same overall dimension and substrate specifications.

## Dual Mode Resonator

**Fig 1:**
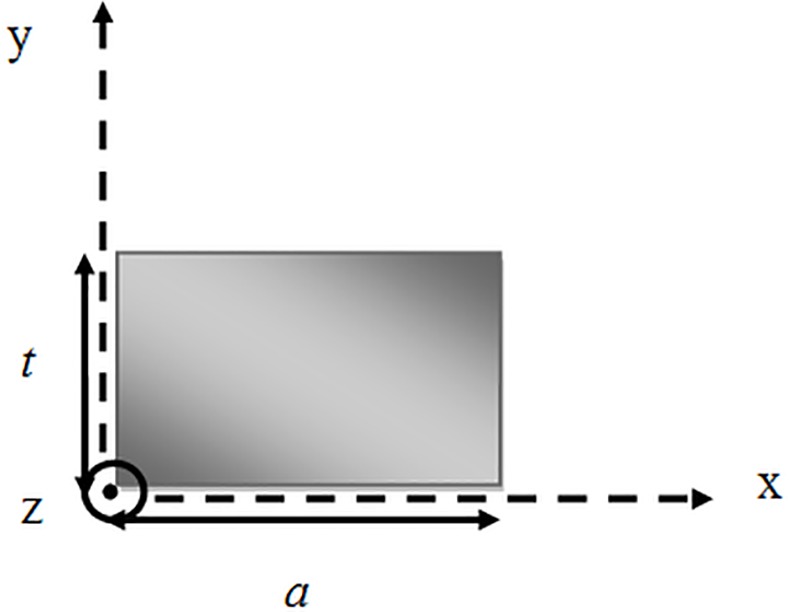
Top view of a generic rectangular patch.

To discuss this concern, let us start with a microstrip rectangular patch resonator top view as in **Fig 1**, represented by a Wheeler’s cavity model [13], that in this case the modes are transverse magnetic *TM*, with the magnetic field orthogonal to z-axis. The electric walls are found perfectly in the upper and lower side of the cavity. On the other hand, the remaining sides are completely magnetic walls.

The EM fields inside the cavity can be defined in terms of $TM_{mn0}^{z}$ modes:

$$E_{z}=A_{mn}\sum_{m=0}^{\infty }\cos\frac{m\pi }{a}x\sum_{n=0}^{\infty }\cos\frac{n\pi }{t}y$$

$$H_{x}=\left(\frac{j\omega \varepsilon_{eff}}{K_{c}^{2}}\right)\;(\frac{\partial E_{Z}}{\partial y})$$

$$H_{y}=-\left(\frac{j\omega \varepsilon_{eff}}{K_{c}^{2}}\right)\;(\frac{\partial E_{Z}}{\partial x})$$

$$K_{c}^{2}={\left(\frac{m\pi }{a}\right)}^{2}+{\left(\frac{n\pi }{t}\right)}^{2}$$ (1)

where *A*_*mn*_ represents the amplitude of mode, *ω* is the radian frequency, *a* is the effective width and *ε*_***eff***_ represents the effective dielectric constant. The resonant frequency of the cavity can be determined by [1, 12]:

$$f_{mn0}=\frac{c}{2\pi \sqrt{\varepsilon_{r}}}\sqrt{{\left(\frac{m\pi }{a}\right)}^{2}+{\left(\frac{n\pi }{t}\right)}^{2}}$$

or

$$f_{mn0}=\frac{1}{2\pi \sqrt{\mu \varepsilon_{eff}}}\sqrt{{\left(\frac{m\pi }{a}\right)}^{2}+{\left(\frac{n\pi }{t}\right)}^{2}}$$ (2)

Actually, there is an infinite number of resonant frequencies related to various field modes or distributions. By analyzing the modes *TM*_100_ and *TM*_010_, it is immediate to prove that the former of magnetic field is directed only towards y axis, while in the latter, it is directed towards x axis. Consequently, the surface current density (*J*_*s*_ = *i*_*z*_ × *H*_*t*_) is oriented only along x in *TM*_100_ while it is oriented along y in *TM*_010_ so that:

$$f_{100}=\frac{c}{\sqrt{\varepsilon_{r}}}\frac{1}{2a}$$

$$\text{and}\;f_{010}=\frac{c}{\sqrt{\varepsilon_{r}}}\frac{1}{2t}$$ (3)

**Fig 2:**
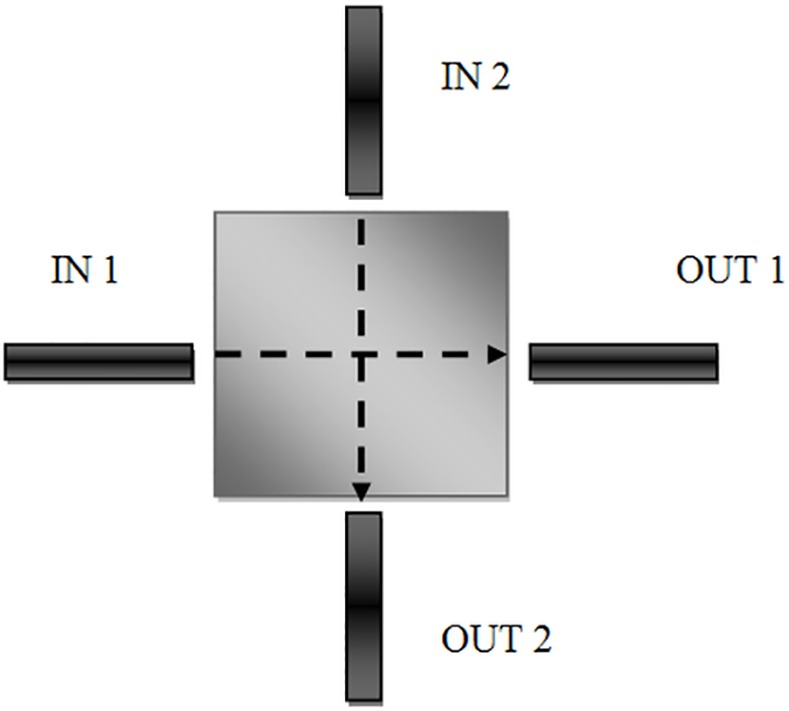
Current distributions of the orthogonal modes in the basic square patch.

In that case, it is apparent that for a square patch (a = t), *TM*_100_ and *TM*_010_ are degenerate modes, since they have identical resonant frequencies and can individually and orthogonally (without power exchange) be energized, as illustrated in **Fig 2**. On the other hand, the modes can be coupled by a sufficient geometry deformation, so that the altered boundary conditions can be fulfilled by the existing two modes. The resultant surface current distribution is then a superimposition of the original orthogonal currents and this gives good reason for the standard placement at the right angle of the feed lines [12].

**Fig 3:**
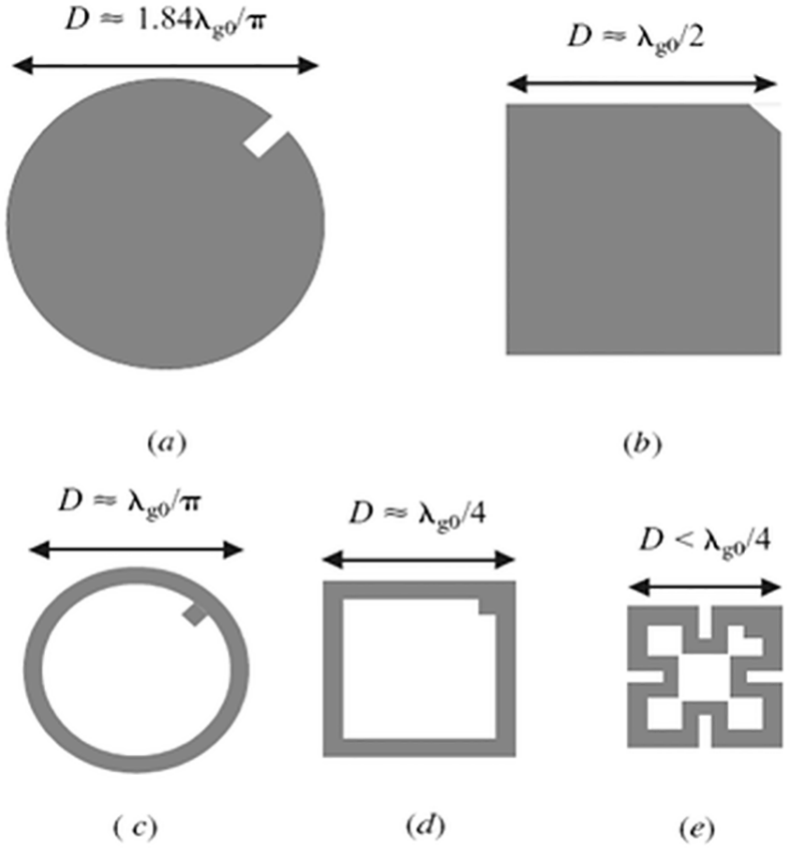
Some microstrip dual-mode resonators (a) Circular disk (b) Square patch (c) Circular ring (d) Square loop (e) Meander loop.

A microstrip dual-mode resonator is not restricted to the square shape, but typically has the planar symmetry. **Fig 3**explains some typical microstrip dual-mode resonators, where *D* in the top of each resonator refers to its symmetrical dimension, and λ_go_ is the guided-wavelength at its resonant frequency in the associated resonator. It is necessary to indicate that a small perturbation has been applied to each dual-mode resonator at offset location that is assumed at a 45° from its two orthogonal modes. For example, a small cut can be used to perturb the square patch and disk resonators, while a small square patch can be inserted into the ring, square loop and meander loop resonators, respectively. Note that for coupling with the orthogonal modes, the perturbations may also take other shapes that are different than those indicated in **Fig 3**. For instance, a small circular disk or patch can be used for coupling the two degenerate modes and, by the same way, a rectangular shape patch can be used instead of square patch for the coupling.

## Slotted Patch Microstrip Filter Design

The bandpass filter requirements generally consist of the specified center frequency, bandwidth, insertion loss value in the passband, and some requisite rejection levels in the stopbands. There will be also a requirement on the return loss magnitude in the passband.

Dual mode microstrip bandpass filter structure has been designed for the resonant frequency of 2 GHz. It has been presumed that this microstrip filter has been modeled using a substrate with a relative insulator constant of 10.8 (Duroid substrate) and 1.27 mm thickness. The guided wave length (*λ*_*g*_) is calculated according to the following equation:

$$\lambda_{g}=\frac{c}{{\text{f}}_{0}\sqrt{\varepsilon_{eff}}}$$ (4)

Where *c* is the velocity of light, *ε*_*r*_ is the relative dielectric constant, f_0_ is the center frequency and *ε*_*eff*_ represents the effective dielectric constant which can be determined from [1, 11, 12]:

$$\varepsilon_{eff}=\frac{\varepsilon_{r}+1}{2}+\frac{\varepsilon_{r}-1}{2}\cdot \frac{1}{\sqrt{1+\frac{12H}{W}}}$$ (5)

**Fig 4:**
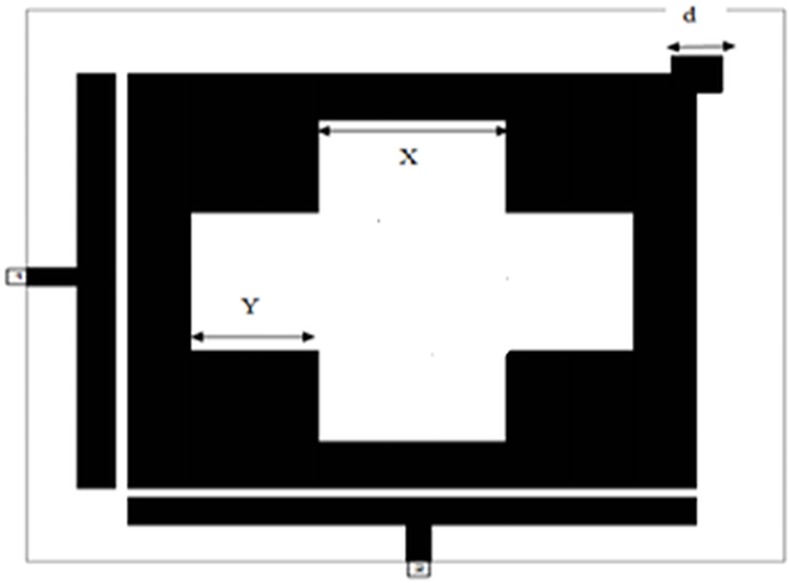
The layout of the modeled dual-mode slotted patch BPF.

*W* and *H* represent the conductor width and substrate thickness respectively. However, the effective dielectric constant can be roughed to (*ε*_*r*_ + 1)/2 [11].The proposed slotted patch bandpass filter is shown in **Fig 4**.

**Fig 5:**
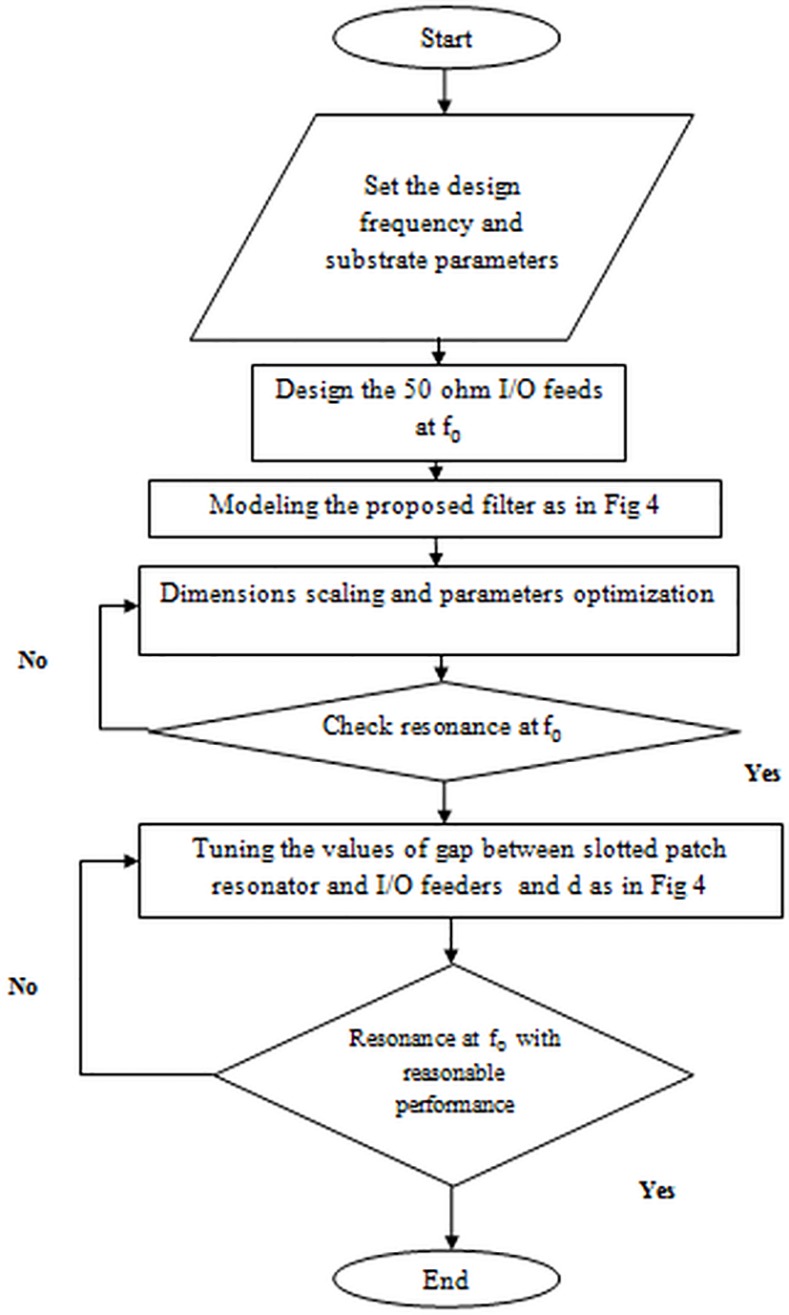
Optimization flowchart for proposed slotted patch filter design.

The procedural steps of the suggested microstrip BPF using Microwave Office EM simulator have been represented in the flowchart as illustrated in **Fig 5**. The dual-mode bandpass filter response can be achieved via the induction of the two degenerate modes by input/output feeders and setting the coupling between the two modes by inserting square perturbation patch in the upper right corner in the resonator [14].

The slot form of the proposed patch resonator acts as somewhat additional perturbation effects to the symmetry of the structure; therefore the field distributions of the degenerate mode will be no longer orthogonal, and they are coupled to each other [14]. This slot is somewhat deformed from 1^st^ iteration of Cantor square fractal geometry depicted in [15].This deformation is used for better resonance tuning and best possible S21 and S11 frequency responses.

The perturbation dimensions of this filter should be tuned for the desired output frequency response, since the coupling strength between the two degenerate modes of the dual-mode resonator is mostly caused by the perturbation dimensions and shape.

## Performance Evaluation

A dual-mode filter structure based on slotted square patch resonator has been modeled and analyzed using Microwave office EM simulator. This simulator carries out electromagnetic analysis using an approach of method of moments (MoM) such that it calculates the filter response by dividing first the resonator in small cell divisions (mesh), adjusted in proportion to the considered necessary accuracy, and then solving a variety of linear equations derived from an integral equation. The cell size here has been chosen to be 0.2 mm. The filter has been executed under frequency range from 1 to 5 GHz with frequency step of 0.001 GHz. Proper boundary conditions are given, and then meshing is carried out on the model to get the final superior mesh. In meshing, it is familiar that more and smaller divisions will give more exact solution. However, these smaller divisions will also require more time for the computer processor to solve the study. For these reasons, it is essential to choose the suitable equilibrium between evaluation time and a satisfactory accuracy level. Using computer devices with four or more core processors can reduce the time of execution in the case of finer mesh. The parametric sweeps solver uses a linear solver algorithm for solution determination.

**Fig 6:**
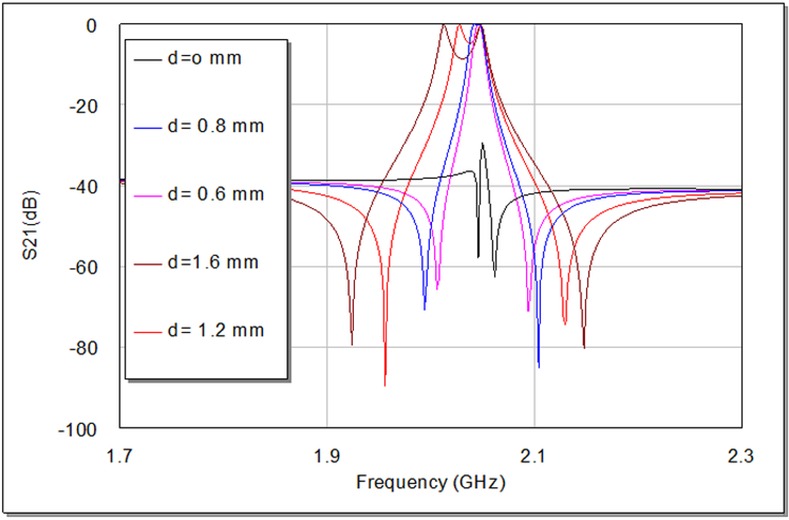
Simulated transmission responses, S21, of proposed BPF as a function of d in units of mm.

**Fig 7:**
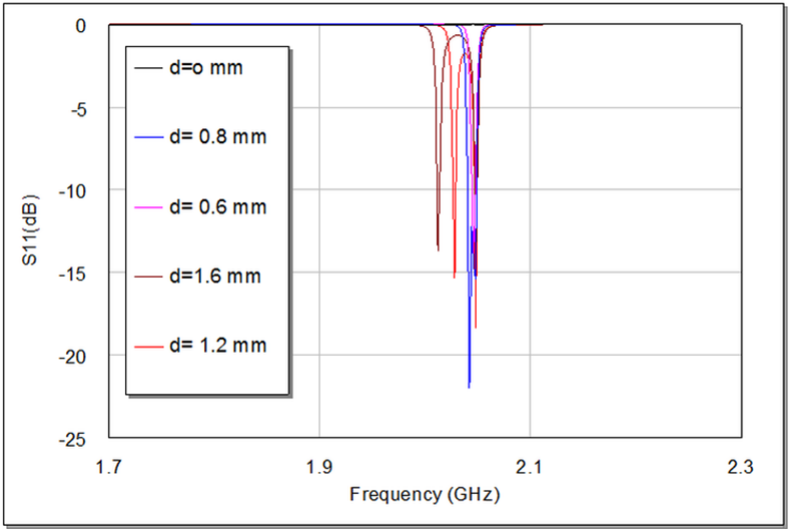
Simulated return loss responses, S11, of the proposed BPF as a function of d in units of mm.

The degree of coupling effect largely depends on the perturbation side length (d) of small perturbation square patch at the right top corner of slotted patch resonator as illustrated in **Fig 4**, which affects the output frequency response of the designed filter [9, 12]. On the other hand, the edge spacing between slotted patch resonator and I/O feeders can be appropriately tuned to maximize return loss and minimize insertion loss to optimize the filter responses as good as possible. A parametric study has been prepared to investigate the effect of d values on the proposed filter response. **Fig 6**and **Fig 7**explain the variation outcomes of S21 and S11 filter responses with respect to different d values.

From **Fig 6**, we can observe that increasing d causes S21 first to move speedily up in the direction of the ideal 0dB point and then split into two observable peaks. Ideally, there would be no coupling between the two modes at d = 0 mm. Table 1 shows the results of the modeled slotted patch filter with d values (0, 0.6, 0.8, 1.2 and 1.6) mm. It is apparent, in **Fig 6**, **Fig 7**and Table 1; the discrepancy in d value slightly influences the resonant frequency, while its effect is more observable on the transmission zeroes, return loss, insertion loss and bandwidth magnitudes. The critical perturbation dimension to generate a satisfactory frequency response can be observed at d = 0.6 mm. Also, the variation in bandwidth is obviously proportional to d values as it is recognizable by Table 1, which can act as controllable parameter for the requested bandwidth in 3 dB passband region.

**Table 1:** Simulation Result Parameters of Slotted Patch BPF With Respect to d Values.

Parameters	d = 0 mm	d = 0.6 mm	d = 0.8 mm	d = 1.2 mm	d = 1.6 mm
S_11_ (dB)	……….	14.21	22	15.32	13.7
Insertion Loss (dB)S21^0^	……….	0.4342	0.1678	-4.62	8.51
Center Frequency (GHZ)	……….	2.042	2.042	2.028	2.012
Bandwidth (MHz)	………	8	12	27	50

By using the principles of previous parameters and adopted design flowchart depicted in **Fig 5**, an optimal microstrip slotted patch BPF at resonant frequency of 2 GHz has the total side length (L) of 18 mm while the perturbation side length (d) is 0.8 mm and the gap between I/O feeders and slotted patch resonator is 0.2 mm. Also, the values of X, Y are 1 mm and 0.6 mm respectively.

**Fig 8:**
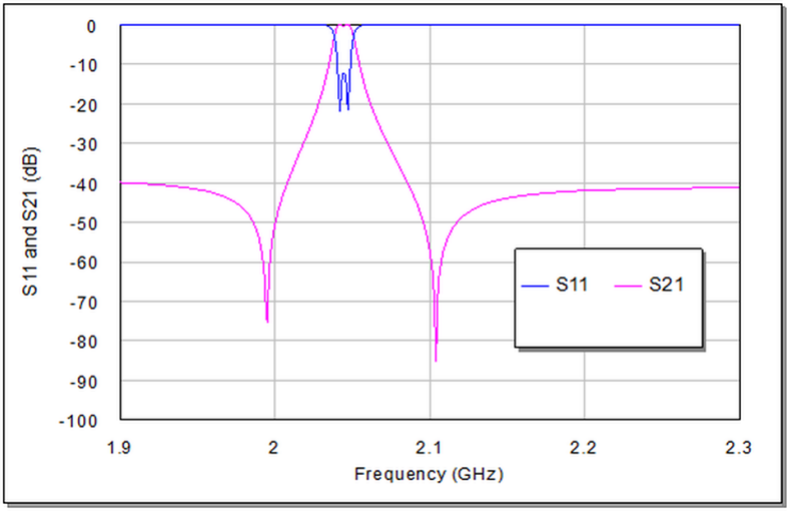
In-band frequency response for microstrip bandpass filter shown in Fig 4.

The finest simulation results from return loss and transmission responses from this filter is shown in **Fig 8**. It can be indicated from this graph that the transmission response has dual transmission zeros that represent band rejection levels as well as two resonances at 2.042 and 2.046 GHz in 3dB passband region. The return loss and insertion loss magnitudes are 22 dB and 0.1678 dB respectively.

**Fig 9:**
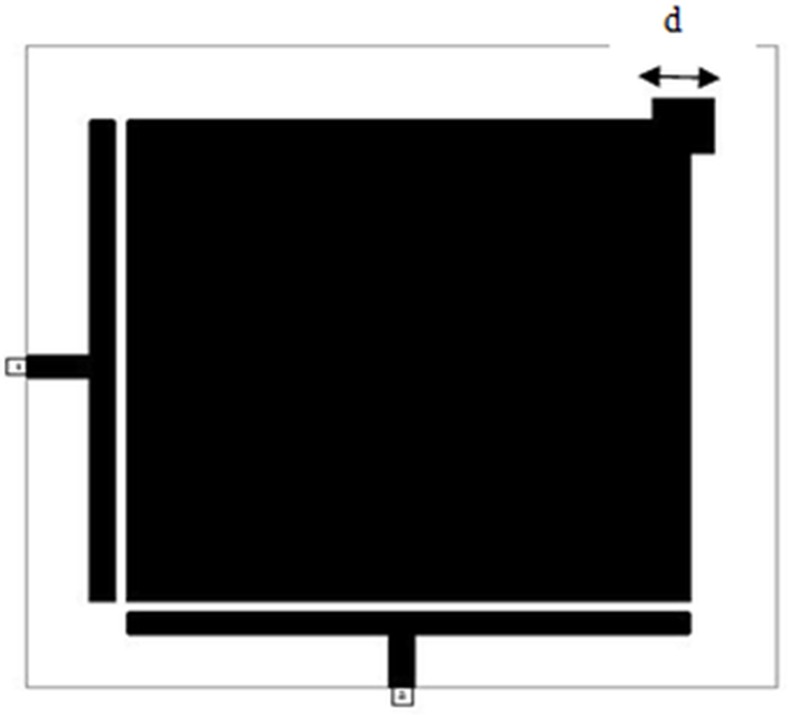
The layout of the modeled dual-mode conventional patch BPF.

**Fig 10:**
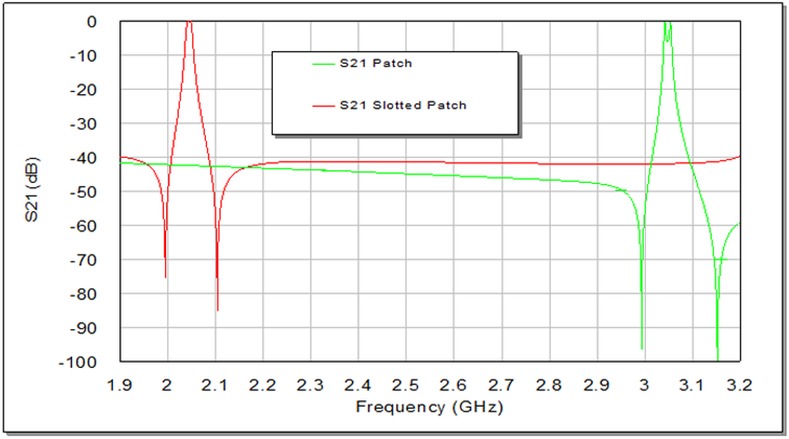
The transmission responses of the two filters; with and without fractal slot.

**Fig 11:**
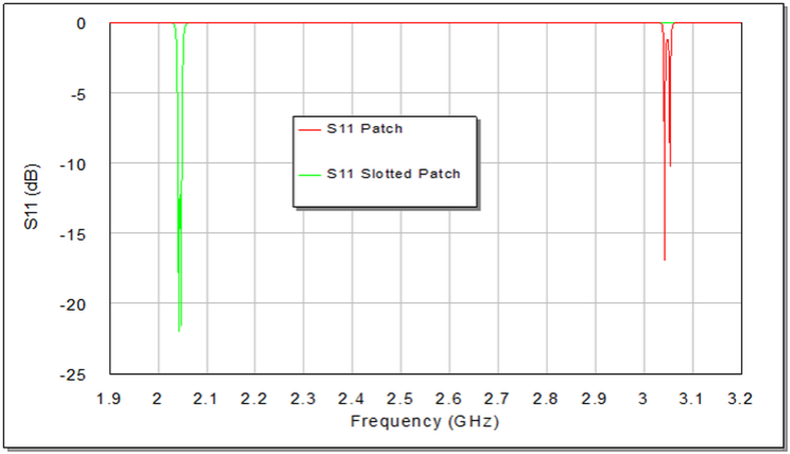
The return loss responses of the two filters; with and without fractal slot.

**Fig 9**illustrates the layout of the dual-mode traditional patch BPF. The use of slotting principle on the traditional patch resonator reduces their fundamental frequency. This is due to the application of surface cuts which increase the current path length and produce a decrease or shifting in the resonance frequency and transmission zeros without changing the external dimensions as it can be seen from S21 responses depicted in **Fig 10**and S11 responses illustrated in **Fig 11**.This acts as miniaturization factor in addition to dual mode property because decreasing the fundamental frequency requires more dimension scaling. Consequently, the decreased fundamental frequency has been obtained by slotting the original traditional patch resonator from 3.042 GHz (without uniform fractal slot) to 2.042 GHz (with uniform fractal slot as in projected design) without the external dimensions change.

Table 2 shows the comparative results of the modeled filter with and without fractal slot corresponding to filter performance parameters and effective areas under same substrate specifications and overall dimensions.The slotted patch filter has noticeable narrower bandwidth as compared to traditional filter based on square patch resonator which represents a big intention in telecommunication systems in order to make the filter capable of declining the interference from strong signals operating in the adjacent bands. Also, it has better insertion loss and return loss values. There is simple degradation in the stopband levels for the slotted patch filter as compared with traditional one. However, these values are very satisfactory in filter design theory.

**Table 2:** Comparative Results between Slotted Patch Filter and the Traditional One.

Filter Dimensions and Parameters	Slotted Patch Filter	Traditional Patch Filter
Overall Area, mm^2^	376.36	376.36
Effective Area, mm^2^	244.36	376.36
Band Rejection levels (dB) (Transmission Zeros Locations)	75(left) 85(right)	96.718(left) 100.71(right)
S_11_ (dB)	22	16.96
Insertion Loss(dB)	0.1678	6.18
Bandwidth (MHz)	12	14.3

One of most serious problems that degrade the bandpass filter performance is the harmonic frequency. Harmonic frequency is a component frequency of filter response that is an integer multiple of the fundamental frequency, for instance, if the fundamental frequency is *f*, the harmonics have frequencies of 2*f*, 3*f*, 4*f*, … etc.

**Fig 12:**
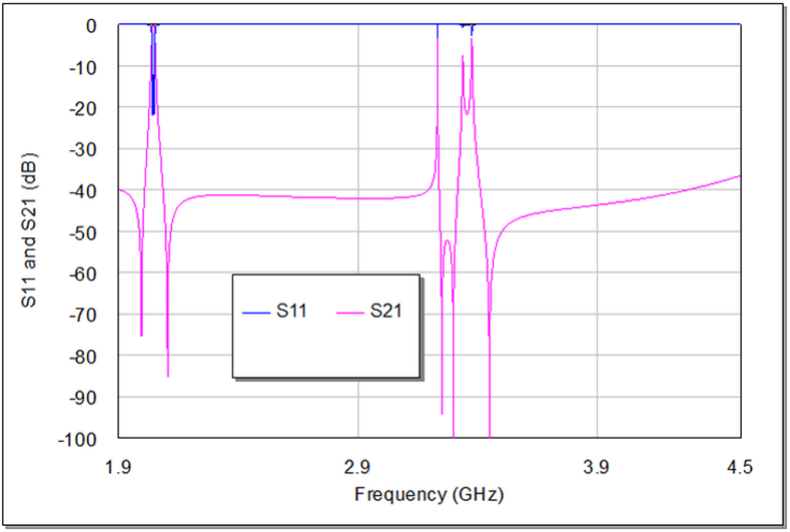
The out-of-band frequency responses of the proposed filter designed around 2 GHz fundamental frequency.

**Fig 12**shows the out-of-band transmission responses of the designed filter at 2 GHz. It is clear that the frequency responses do not sustain second harmonic frequency that typically comes with the bandpass filter responses. There are some spurious responses appearing in this figure, however, they are not effectual since they have very narrow bandwidth and low return loss magnitudes.

**Fig 13:**
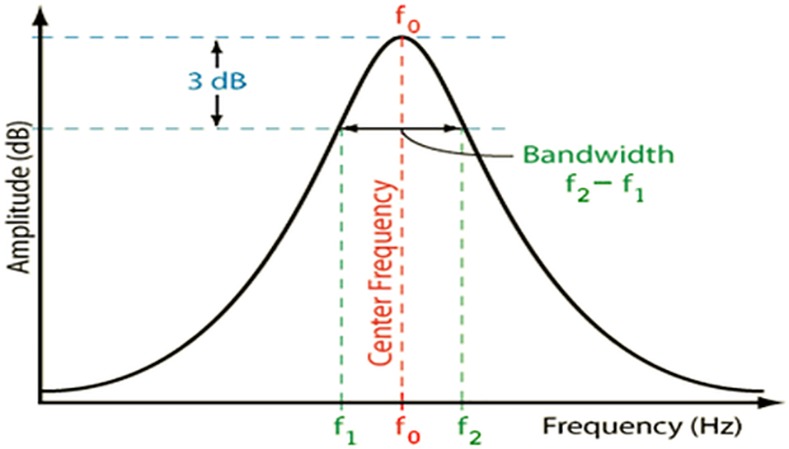
Basic transmission response of BPF and inband frequencies.

The coupling coefficient can be calculated generally from in-band frequencies, f_1_ and f_2_ as in **Fig 13**by [12]:

$$\text{K}12=\frac{2({\text{f}}_{2}-{\text{f}}_{1})}{\left({\text{f}}_{2}+{\text{f}}_{1}\right)}$$ (6)

On the other hand, the external quality factor can be extracted from 3dB bandwidth transmission peak by [12]:

$$\text{Qex}=\frac{2{\text{f}}_{ο}}{{\text{BW}}_{3\text{dB}}}$$ (7)

**Fig 14:**
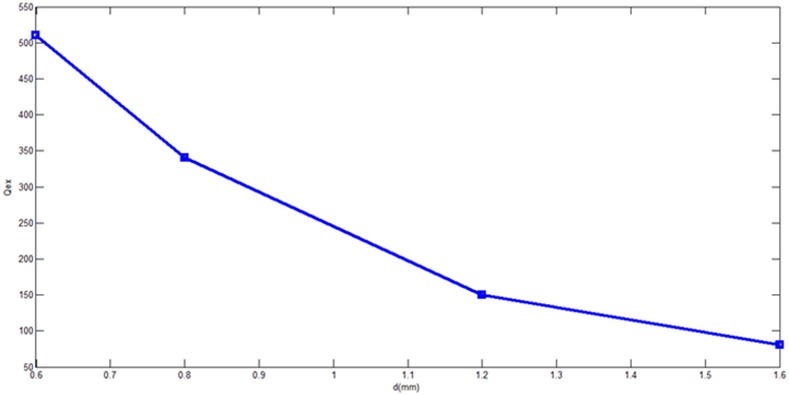
External quality factor Qex as function of d.

**Fig 15:**
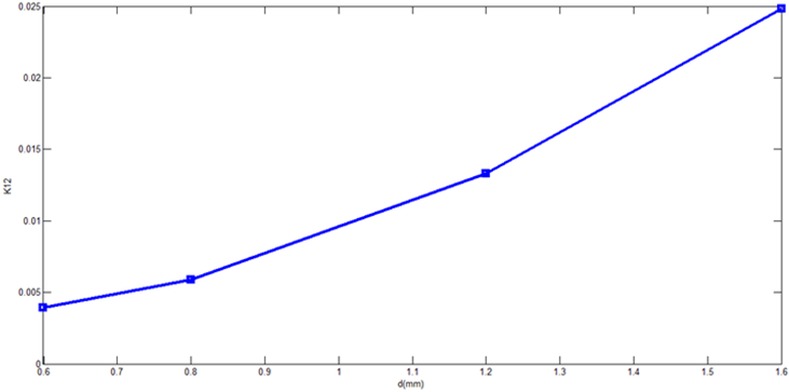
Coupling coefficient K12 as function of d.

The external quality factor Qex decreases as d parameter increases while the coupling coefficient K12 increases from increasing the perturbation side length d as it can be concluded from **Fig 14**and **Fig 15**respectively for different d values ranged from 0.6 mm to 1.6 mm. In the other words, the d value determines the external quality factor and coupling factor magnitudes effectively since it changes the 3 dB bandwidth distinctly.

The planned slotted patch filter for recent wireless applications has observable small size, less insertion loss, narrower bandwidth and good return loss magnitudes than simulated filters reported in [16, 17] under same centre frequency as it can be predicted from Table 3.

**Table 3:** Comparasion of the Proposed BPF for Wireless System with [15] and [16] at Same Center Frequency.

Filter Parameters	Our Work	[15]	[16]
Insertion Loss (dB), S21	0.1678	1.1	2.343
Return Loss (dB), S11	22	22	26.455
Overall dimension (mm^2^)	376.36	695.81	347.375
Bandwidth (MHz)	12	100	26.4

## Conclusion

A new dual degenerate mode microstrip bandpass filter has been proposed in this study. This filter uses microstrip patch resonator with geometrical slot based on Cantor square fractal geometry. The filter structure has been determined using Microwave Office EM simulator. It has been designed at resonant frequency of 2 GHz using a substrate with a dielectric constant of 10.8 and thickness of 1.27mm.Performance simulation results show that the designed filter offers high-quality frequency responses as well as narrow band gained, compactness properties and blocked 2^nd^ harmonic in out of band region.

An extensive comparative study for the proposed filter in accordance with bandpass filter based on traditional square patch resonator has been carried out using same overall dimensions and substrate specifications. Also, a parametric study about the effect of perturbation side length (d) on the slotted patch filter in terms of electrical parameters has been extracted from this research article. From these parametric investigations, it is found that the bandpass filter using geometrical fractal slot has better frequency response in terms of insertion loss, return loss and bandwidth magnitudes as well as the decreased resonant frequency as compared with the traditional square patch filter. Also, it is found that the perturbation dimension hugely determines the electrical specifications for the adopted slotted patch bandpass filter.
